# The Mental Health Literacy and Stigma Scale—*Bilingual* Cultural Adaptation: Validity and Reliability Pilot Study in Nursing Students

**DOI:** 10.1002/nop2.70073

**Published:** 2025-01-17

**Authors:** Maria José Nogueira, Síria Sas, Lucía Rodríguez, Andrea Carbonero, Uxía Bello, Leandro Nascimento, Susana Mendonça, Delfina Teixeira

**Affiliations:** ^1^ Nursing Departemant Évora University Évora Portugal; ^2^ Nursing Departement Center for Research in Health Technologies and Services Portugal; ^3^ Nursing Complejo Hospitalario Universitario de Ourense Ourense Spain; ^4^ Nursing Departement Institut Politécnic of Santarém Santarém Portugal

**Keywords:** literacy, mental health, nursing students, psychometric testing, reliability, stigma, validity

## Abstract

**Aim:**

To describe and evaluate the psychometric properties (reliability and construct validity) of the Mental Health Literacy and Stigma Scale‐*Bilingual* (MHLaSS‐B).

**Design:**

This is a methodological study designed in a convenience sample of 271 Portuguese and Spanish nursing students who volunteered to participate in the research.

**Methods:**

The Mental Health Literacy and Stigma Scale‐*Bilingual* version (Spanish and Portuguese) was used for data collection. MHLaSS‐*B* intercultural adaptation followed three stages: translation, back‐translation and pilot test and the *thinking‐aloud* techniques. Content validity was performed by Content Validity Index and Modified Kappa statistic. Construct validity and reliability tests were performed. Internal consistency was assessed by Cronbach's alpha. Data were analysed using SPSS programs. Ethics committee approval and permission from the institution involved were obtained.

**Results:**

The MHLaSS‐B has 28 items of one‐factor structure. Cronbach's alpha was 0.702. The cumulative variance explained was 23.14%. Respondents show *High literacy* and *Low Stigma* (*M* = 23.4).

**Patient or Public Contributions:**

The availability of the MHLaSS‐B allows the general population of Portugal and Spain to have a versatile instrument for assessing stigma and MHL. The MHLaSS‐B presents good psychometric properties, and it can be a useful tool for novice health workers to better understand the aspects they should pay attention to manage MHL and stigma successfully. The MHLaSS‐B is a reliable, adaptable instrument that is now available and it can be used in investigation, teaching and clinical practice.

## 1 Introduction

Stigma towards mental illness along with mental health literacy (MHL) deficits are responsible for the poor rates of mental health professional help (Simões de Almeida et al. 2023) and remain a social challenge in contemporary societies (Cheng et al. 2018). Overall, it is the current barrier to help‐seeking (Sastre‐Rus et al. 2019) including health workers and college students from different course areas (Ebert et al. 2019; Querido, Tomás, and Carvalho 2016; Vogel et al. 2017). In addition, research shows that the general public has quite poor recognition of the symptoms of mental health disorders and consistently shows that better levels of MHL are related to better health outcomes (Sickel, Seacat, and Nabors 2019). Also, a direct relationship between MHL levels and stigma has been established, in several populations including health workers and students (Bitzer and Sorensen 2018; Mackert et al. 2019; Reavley, McCann, and Jorm 2012). MHL has been identified as a factor influencing early help‐seeking for mental health problems and stigmatising attitudes (Soria‐Martínez et al. 2023).

Stigma is a form of violence, often defined as a ‘mark of shame’ or ‘disgrace’ resulting in exclusion (Amaechi, Nwani, and Akadieze 2023). The negative consequences of stigmatising attitudes include being rejected, discriminated and excluded from participating in several areas of society (Amaechi, Nwani, and Akadieze 2023). Considering the steadily increasing prevalence of mental disorders stigma presents itself as a public health challenge, particularly in measuring and designing accurate interventions to improve mental health literacy (MHL), reduce mental health stigma and promote mental well‐being (Ma, Anderson, and Burn 2023; Simões de Almeida et al. 2023).

Literature shows the fight against mental illness stigma has increased in recent years in the overall population, health workers and college students, endorsing the need to increase mental health literacy levels (Amaechi, Nwani, and Akadieze 2023). Additionally, for undergraduate students, from different areas of health (social work, medicine and nursing), addressing stigma and MHL as a syllabus is mandatory, once students must learn and expand their theoretical knowledge for subsequent application in Clinical Teachings (Sandhu et al. 2019). This strategy promotes the transferability of skills and knowledge and seems to be effective because health students interact with different patients and care contexts, which includes dealing with different users with mental illnesses and needs. Therefore, as future health workers, students must learn and develop skills to assess and deal with mental health stigma (Simonelli‐Muñoz et al. 2023), and improve and promote MHL. This knowledge and training are essential to provide care with the greatest possible neutrality, without discriminating, often rejected by society, as well as to spread and contribute to the normalisation and destigmatisation of people with mental illness (Amaechi, Nwani, and Akadieze 2023; Vidourek and Burbage 2019).

Evidence shows a direct relationship between stigma and health literacy level and a relationship between inadequate literacy levels and implications for individual and collective health, with better health literacy levels being associated with better health outcomes (Gallego et al. 2020; Pedro, Amaral, and Escoval 2016). However, it has been a challenge for health workers, teachers and society in general, to find efficient instruments and strategies to face discrimination against people with mental illness (DeBate, Gatto, and Rafal 2018; Samari et al. 2018; Vidourek and Burbage 2019).

Furthermore, the most effective strategies to promote help‐seeking behaviour, reduce stigma and improve confidence in providing help to individuals showing signs of a mental health disorder are grounded on mental health literacy. MHL refers to the beliefs and knowledge about mental problems and disorders that allow recognition, management (in the sense of self‐care) and prevention. Inadequate levels of MHL yield individual and collective health negative implications (Aldalaykeh, Al‐Hammouri, and Rababah 2019; Jorm 2000; Reavley, McCann, and Jorm 2012). Thus, currently, MHL is a central concept for health workers' knowledge and is widely used as a powerful intervention (Gallego et al. 2020; Loureiro and Sousa 2019; Morgan et al. 2019). The concept includes three outsets: the knowledge that benefits mental health and the skills to provide a person's first aid (Jorm 2000); alludes to stigma, historically considered separately from literacy; and extends Jorm's concept of self‐help strategies by including seeking effective help (Mackert et al. 2019).

In addition, research consistently shows that the general public has quite poor recognition of the symptoms of mental health disorders and better levels of MHL are related to better health outcomes (Sickel, Seacat, and Nabors 2019). In students, a direct relationship between mental health literacy levels and stigma has been established (Bitzer and Sorensen 2018; Mackert et al. 2019; Reavley, McCann, and Jorm 2012).

Despite being a challenge, measuring MHL and the stigma is essential (Corrigan et al. 2016; Kosyluk et al. 2016; Sastre‐Rus et al. 2019), but to do so, valid and reliable instruments are needed for clinical practice, teaching and research purposes (Sastre‐Rus et al. 2019).

Globally, the literature shows that there is a lack of instruments to measure MHL and stigma, as in Portugal and Spain. Nevertheless, it was possible to list some instruments that are in use in these two countries: the *Australian National Survey of Youth and Parents* (Jorm, Wright, and Morgan 2007); *Spanish version of the Mental Health Literacy Scale (MHLS)* (Castellvi et al. 2019); the *Attribution Questionnaire* (Corrigan et al. 2001); the *Attitudes to Mental Illness Questionnaire* (Luty et al. 2006); the *LSMq‐young adults* (Dias et al. 2018); and the *Mental Health Literacy Assessment Questionnaire* (QuALiSMental) (Loureiro 2018). Yet, all these instruments measure Stigma and MHL concepts separately and most of them are very extensive, which is a disadvantage that limits their use, as they are less appealing because it requires more time to complete. Additionally, no instrument was found that covers MHL and Stigma constructs at the same time, nor in Portuguese or Spanish. Novel and creative approaches may increase the capacity to target wide populations in two different cultural contexts and languages. Versatile instruments to measure stigma gather several advantages to face stigma‐reducing challenges. A valid, reliable and succinct measure is a useful tool for wider purposes like community education, early recognition, training and research (Chang et al. 2017; Mackert et al. 2019; Vogel et al. 2017).

Thus, we find it relevant to validate the bilingual version of the *Qué Sabes sobre la enfermedad mental? In* Portuguese and Spanish. A bilingual instrument can be used in the general population in both countries and as a useful didactic instrument. This kind of instrument can be useful for students training to implement anti‐stigma programmes and for individual and collective screening for MHL and mental illness‐related stigma (Gallego et al. 2020). The above‐mentioned limitations of previous measures, support the need for an up‐to‐date assessment of this construct. Instruments´ cultural adaptation is essential for the test's integrity and to become understandable and relevant for the new language and population (Marôco et al. 2014). Thus, any test that lends itself to validation and, subsequently to use, must be the result of these standardised analyses (Marôco et al. 2014).

This study aims to translate, describe and evaluate the psychometric properties (reliability and validity) of the Mental Health Literacy and Stigma Scale *Bilingual* (MHLSS‐*B*), in Portuguese and Spanish nursing students.

### 1.1 Design

A quantitative approach and a methodological study design were used on a Portuguese and Spanish sample of nursing students who volunteered to participate in the research. The quantitative approach focuses on measuring variables and testing hypotheses or theoretical models using numerical data (Cathala and Moorley 2018), while the methodological study aims to develop, test or refine research instruments, procedures or techniques. Thus, to validate the MHLaSS‐B, and ensure that it is accurate and reliable, we consider these approaches appropriate (Marôco et al. 2014). This pilot study comprised two phases: (1) Intercultural adaptation of the MHLaSS‐*B* to Portuguese; (2) a scale evaluation (evaluation of psychometric properties of MHLaSS‐*B*, following the methodological principles (Marôco et al. 2014)).

## 2 Materials and Methods

The data to test the psychometric properties (reliability‐internal consistency and construct validity) of the MHLSS‐*B* were collected using an online survey of Portuguese and Spanish nursing students, during the first semester of 2020.

### 2.1 Phase 1: Intercultural Adaptation of the MHLaSS‐*B*

The first phase of the intercultural adaptation of the original Spanish version of the Mental Health Literacy and Stigma Scale process into Portuguese followed the standard procedures of the methodological process of instruments in health (Farrés‐Tarafa et al. 2020; Henriksen et al. 2020; Memon et al. 2017). We follow the major three stages suggested: translation, *back‐translation* and pilot test (*n* = 10) (Devine et al. 2018). The MHLaSS‐*B*, was originally called *Qué Sabes Sobre la Infermedad mental?* a self‐report questionnaire with 28 items (1–9 about mental health literacy and 10–28 about mental health stigma, created by the association *Aragón sin stigma* in 2019 (Aragonese Mental Health Entities of the Zaragoza City Council 2019a) to identify the population's level of knowledge and stigma about different mental illnesses.

#### 2.1.1 First Step—Translation

The Spanish questionnaire *qué sabes sobre la enfermedad mental*? (Aragonese Mental Health Entities of the Zaragoza City Council 2019a) was translated from Spanish into Portuguese to obtain conceptual and linguistic semantic equivalences. Two Portuguese native speakers, bilingual in Spanish, independently translated the questionnaire into Portuguese. A collaborative pooled version was then obtained from the two translations to create a consensus version.

#### 2.1.2 Second Step—Back‐Translation

The pooled version was back‐translated into Spanish by two Spanish native speakers' translators bilingual in Portuguese. A Spanish version draft was obtained from the comparison between the original and the back‐translation instrument.

#### 2.1.3 Third Step—Face and Content Validity

Face validity was carried out to analyse the items agreed upon in the previous phase, through three stages: expert review, scoring and achieving consensus (Roy et al. 2023). Face validity represents the extent to which a test appears to measure what it is intended to measure with no ambiguity (Wynd, Schmidt, and Schaefer 2003). The experts reviewed and rated the face MHLaSS‐B validity of each item on a scale of 1–4, for understanding, ambiguousness, clarity and chance of misinterpretation. Average scores were calculated for each item to determine the overall face validity score, according to the criterion proposed by Roy et al. (2023), a mean ≥ 3 is accepted.

Content validity is the degree to which the instrument covers all items necessary or sufficient to measure the construct of interest or the degree of representativeness (Wynd, Schmidt, and Schaefer 2003). Seven mental health experts (similar to face validity experts' profile) rated items using a priori criteria (clarity, relevance and representativeness of the items), using a ‘4‐response’ scale (3 = must be required, 2 = Required, 1 = Useful, but not required and 0 = Not useful, must be removed). The Content Validity Index (I–CVI) were used to measure content validity. For the calculation of I–CVI (Item level), the response categories for each item were dichotomised by combining responses 3 and 2 as ‘relevant’ and 1 and 0 together as ‘not relevant’. Higher proportion agreement ratings might be due to random chance, so a complement analysis was performed using a multi‐rater kappa coefficient of agreement. Kappa statistic is an important supplement to CVI since provides information about the degree of agreement beyond chance. Modified Kappa statistic (*K*) was computed and agreement was categorised based on the *K* values (Wynd, Schmidt, and Schaefer 2003). The experts' consensus about ambiguous, unclear or unusual wording was achieved using group discussions to explain the experts' reasoning and to compromise the final solution.

#### 2.1.4 Fourth Step—Pilot Test

A pilot test was performed in the Portuguese and Spanish draft versions in 10 Portuguese and Spanish‐speaking nursing students. The authors conducted group discussion meetings using *thinking‐aloud* techniques to safeguard the clarity and unbiased of the questions of the item (Devine et al. 2018; Farrés‐Tarafa et al. 2020). Students had paper‐printed copies, and were required to answer two questions for each item: ‘*What does this statement mean to you*?’ and ‘*Is there any other wording that enables this meaning to be expressed more clearly*?’

### 2.2 Phase 2: Scale Evaluation of the MHLaSS‐Bilingual

The second phase consisted of the study and analysis of the psychometric properties—reliability (internal consistency), and construct validity of the MHLaSS‐*B* (Portuguese and Spanish), following the methodological principles (Marôco et al. 2014).

### 2.3 Participants and Setting

The study sample consisted of 271 Portuguese and Spanish nursing students. A non‐probability convenience sampling was used, recruiting during the 2020/2021 academic year (first semester) in a nursing school north of Portuguese, near the Spanish border with both nationalities of students. The sample size was calculated using the rule of 5–10 participants for each item in the assessment tool according to the recommendations of Streiner, Norman, and Cairney (2015). It was defined as a minimum mandatory number of 100 participants to fulfil the criteria to carry out an exploratory factor analysis. The inclusion criteria were as follows: (a) to be a current student of the Portuguese nursing school; (b) to be a Portuguese or Spanish native speaker; and (c) to be aged over 18 years. Using the campus email, all students were invited to fill out an anonymous online survey, comprising all variables of the study (Streiner, Norman, and Cairney 2015).

### 2.4 Variables, Measures and Information Source

The online questionnaire has two parts: (1) demographic information using five questions (sex, age, nationality, residence status and academic year); (2) The Mental Health Literacy and Stigma Scale – *Bilingual* (MHLaSS‐B) (Table 2). The MHLaSS‐B identify the population's level of knowledge and stigma about different mental illnesses. It is a self‐report instrument with 28 items that include nine questions about mental health literacy (items 1–9) and 19 questions about mental health stigma (items 10–28). The MHLaSS‐B answers are multiple‐choice with three options (a, b or c). The respondents mark only one correct answer, which best reflects their perceptions or personal circumstances. For each correct answer, one point is assigned and for each incorrect answer zero points. The total MHLaSS‐B score ranges from 0 to 28 points, and higher values correspond to better literacy and less stigma towards mental illnesses. Qualitative analysis of the MHLaSS‐B is possible to consider three cut‐off points: *High Stigma Low literacy* (0 and 11 points)—corresponds to high stigma, feelings of fear and distance from a person with mental illness, classified as dangerous due to the lack of knowledge of the respondent; *High literacy medium Stigma* (12 and 22 points)—classifies the participant's knowledge as relatively high, but the level of stigma is sufficient to cause discomfort or avoid contact with people with mental illness; and *High literacy* and *Low Stigma* (23 and 28 points)—place the level of the stigma of the participant at the minimum and with high knowledge about the mental illness.

### 2.5 Statistical Analysis

Statistical analysis was performed using the *Statistical Package for the Social Sciences* IBM SPSS version 23.0. The sample characteristics were analysed using descriptive statistics reporting *n* (%) for categorical data. For construct validity, an Exploratory Factor Analysis (EFA) was performed on the structure of the MHLaSS‐B. The adequacy and normality test of the sample was performed by Kaiser–Meyer–Olkin (KMO) and Bartlett's test of sphericity was performed for relevance analysis of the sample (Hacer and Yildirim 2020). For the extraction of the factors, we assumed: the Kaiser rule (the components with an eigenvalue > 1 were retained); the scree plot graph was inspected (factors above the curve were retained); the commonalities and inter‐items' matrix coefficients > 0.40 were taken as significant (Ferrando and Lorenzo‐Seva 2018; Watkins 2018). Reliability was analysed by internal consistency calculated by Cronbach's alpha coefficient test. The test–retest reproducibility was evaluated using the intraclass correlation coefficient (ICC), and the value of 0.65 was accepted as adequate reliability for a group of patients.

### 2.6 Ethical Considerations

The study protocol was registered and approved by (P07/2020 ESECVP‐AT) and the authorisation to use and translate the scale was obtained. Also, the answer's confidentiality and the right to drop out at any moment with no consequences were assured. All participants were informed by email about the purpose of the study, that no financial charges or risks were involved and that they freely gave their written consent to participate in the study as volunteers. A code was attributed to each participant. The address of the principal investigator was also provided to solve any potential doubt.

## 3 Results

### 3.1 Respondents Characteristics

A total of 271 Portuguese and Spanish nursing students completed the study, mostly females (82.94%) aged between 18 and 54 years old (*M* = 26.2; SD = 8.5), the majority (40%) were freshmen and displaced from home (71.6%). All participants were undergraduate students and 38.22% (third year and senior students) had previously attended classes related to Mental Health Literacy and Stigma (included in the curriculum syllabus). Table 1 presents the sociodemographic details and characteristics of the participants. Respondents show *High literacy* and *Low Stigma* (*M* = 23.4).

**TABLE 1:** Demographic characteristics of the sample (*N* = 271).

Variables	*N*	%
Gender		
Female	242	82.94
Male	29	17.05
Age		
18–25	211	65.30
26–35	26	14.70
36–54	34	20.00
	(*M* = 26.2; DP = 8.5)	
Nationality		
Portuguese	127	41.7
Spanish	144	58.3
Residence status		
Not displaced	50	28.4
Displaced	221	71.6
Academic year		
Freshman	112	40.02
Second	57	21.76
Third	40	14.11
Senior	62	24.11

### 3.2 Intercultural Adaptation of the MHLaSS‐*B*

Results from the Intercultural adaptation show that the MHLaSS‐*B* obtained consensus in the *back‐translation* version, clarity and unbiased of the items on *thinking‐aloud* group techniques and in the *pilot test* participants concluded that it was easy to understand and required little time (5–10 min) for scale completion. The instrument was named as MHLaSS‐*Bilingual*.

Results from the descriptive analysis of the MHLaSS‐B show that the majority of the participants scored at High Literacy and Low Stigma (23 and 28 points) with a total mean of 23.4 and SD = 3.2. Item 14 obtained the lowest score and items 2 and 19 were the highest. Results also show that the stigma decreases as literacy increases and a significant difference (*p* < 0.01) was found in stigmatising attitudes between students with different levels of knowledge. Students with theoretical training and internship scored less stigma (*M* = 25; SD = 3.5) than students with no theoretical training or only theoretical training (*M* = 22; SD = 3.0).

### 3.3 MHLaSS‐B Face and Content Validity

Overall, MHLaSS‐B has good face validity. The face validity averages for MHLaSS‐B were 3.51, with scores of 3.72, 3.41, 3.67 and 3.25 for understanding, unambiguity, clarity and less chance of misinterpretation, respectively (all items had a mean score ≥ 3).

The MHLaSS‐B Content Validity Index (CVI) is acceptable (CVI = 0.829). The I–CVI for MHL (items 1–9) and Stigma (items 10–28) varied from 0.45 to 1 and from 0.6 to 1, respectively. For the MHL assessment, six items (MHL 1, 2, 3, 4, 6 and 8) obtained the highest score of 1, and six items (55.5%) had I–CVI values > 0.78, which is appropriate. Three items (33%, MHL 5, 7 and 9) needed revision (prepositions and synonyms). For the stigma assessment, seven items (S 11, 12, 14, 17, 19, 20 and 25) obtained the highest score of 1. Out of 18 items of Stigma, 12 items (63.1%) had I–CVI values > 0.78 which is appropriate. Two items (10%, S 13 and 22) needed revision (prepositions). Five items scored low kappas (from 0.30 to 0.40) indicating fair agreement among the experts. Item‐wise modified kappa statistics of MHL and Stigma ranged between 0.30–1 and 0.80–1, respectively. Out of nine MHL items, seven items (77.7%) had a ‘Substantial’ agreement and two (22.3%, MHL 5, and 9) had a ‘Fair’ agreement. Out of 19 Stigma items, 15 items (78.9%) had agreement ranging between ‘Moderate’ and ‘Substantial’. Two items (10.5%, S 13 and 22) had a ‘Fair’ agreement. Based on the Content Validity Index and Modified Kappa Statistics, the MHLaSS‐B elicited overall acceptable Content Validity.

Experts diverged slightly in ratings, so the wording of items 5, 7, 9, 13 and 22 (mostly prepositions or synonymous) were rephrased to improve simplicity in the sentence and to adjust the items with close meaning, enabling them to be expressed more clearly. The final consensual wording of the MHLaSS‐B is presented in Table 2.

**TABLE 2:** MHLaSS‐B mean and standard deviation of items and total (*N* = 170).

MHLaSS‐B items	*M*	SD
1. What is mental illness?	0.92	0.27
2. Who can affect this disease?	0.98	0.15
3. Between mental illness and intellectual disability	0.95	0.21
4. Which of these disorders is not a mental illness?	0.92	0.27
5. Having a mental illness is the same as	0.87	0.34
6. A person with mental illness	0.44	0.50
7. Mental illness is	0.81	0.40
8. A person with schizophrenia	0.56	0.50
9. Mental illness means	0.85	0.36
10. When you have a relationship with a person with a mental illness, what is your reaction?	0.73	0.45
11. In a job interview, what difficulties do you think a person with mental illness has?	0.72	0.45
12. A person diagnosed with severe mental illness is:	0.64	0.48
13. A person with mental illness is	0.69	0.47
14. Do you consider that a person with a mental illness is dangerous?	0.44	0.50
15. Medication is	0.95	0.23
16. Do you think that a person with mental illness	0.94	0.25
17. If you go on the tram and see a person talking to himself and saying inconsistencies, what do you do	0.89	0.31
18. If someone you know has been diagnosed with a mental illness	0.98	0.15
19. What do you think is the reason why mental illness causes rejection by society?	0.96	0.20
20. What image do the media give of people with mental illness?	0.89	0.31
21. What is the main obstacle to the integration of people with severe and persistent mental illness?	0.83	0.38
22. Where do you think a person with mental illness should be?	0.92	0.27
23. People suffer from mental illness because	0.98	0.13
24. What are the chances of contracting a mental illness?	0.80	0.40
25. Have Mental illness is like	0.85	0.36
26. The mental illnesses are from	0.95	0.21
27. People with mental illness can have	0.92	0.28
28. If you are in a meeting where there is a person with a mental illness	0.85	0.36
Total MHLaSS‐B	23.4	3.2

Construct validity was examined by conducting an items analysis of the MHLaSS‐B that assesses how well a set of scale items matches the relevant content domain of the construct that it is trying to measure. The Kaiser–Meyer–Olkin, a measure of sampling adequacy test, previously verified the adequacy (KMO = 0.694; *p* < 0.5) of the sample for the Exploratory Factor Analysis (EFA). Bartlett's test of sphericity was significant (*χ*2 = 982.129, *df* = 378, *p* < 0.000), indicating significant relationships between the items to allow the EFA. The factor structure of the 28‐item MHLaSS‐B was conducted using Principal Component Analysis followed by a Varimax orthogonal rotation. The first‐order (unifactorial) structure eigenvalue = 4.47, represents a total variance of 23.14% (Table 3). Results suggest the presence of a predominant factor that encompasses a final solution of 28 items of MHLaSS‐B's unifactorial structure (Table 3).

**TABLE 3:** Analysis of MHLaSS‐B, items, eigenvalues, variance and factor loadings (*N* = 271).

MHLaSS‐B items	Corrected item‐total correlation	*α* if the item deleted	Factor loading
1	0.226	0.696	0.552
2	0.383	0.693	0.553
3	0.303	0.693	0.604
4	0.284	0.692	0.655
5	0.329	0.688	0.680
6	0.123	0.708	0.727
7	0.221	0.696	0.632
8	0.044	0.725	0.672
9	0.221	0.696	0.603
10	0.153	0.703	0.603
11	0.111	0.707	0.547
12	0.224	0.697	0.564
13	0.388	0.680	0.712
14	0.285	0.691	0.654
15	0.436	0.686	0.638
16	0.411	0.686	0.661
17	0.373	0.685	0.561
18	0.270	0.696	0.569
19	0.368	0.691	0.680
20	0.258	0.693	0.659
21	0.123	0.704	0.651
22	0.327	0.690	0.598
23	0.336	0.695	0.782
24	0.207	0.697	0.511
25	0.204	0.697	0.680
26	0.376	0.690	0.669
27	0.434	0.683	0.474
28	0.258	0.693	0.585
Eigenvalue		4.47	
Total variance (%)		23.14%	
Total *α* = 0.702			

*Note:* Extraction method: Principal Component Analysis.

### 3.4 MHLaSS‐B Exploratory Factor Analysis Validity

To determine the number of items and the underlying factor structure of the scale, an exploratory factor analysis (EFA) was performed. To study validity, we used the EFA analysis and a unidimensional model seems plausible, based on the bivariate correlation performed, using the statistical technique of principal components analysis. Convergent validity or commonalities (items belonging to a common construct), all extracted factors should exhibit loadings of 0.60 or higher (called same‐factor loadings) and the results show that all items have something in common (values > 0.6) except for item 27 which has =0.474, indicating a single‐factor structure of the MHLaSS‐B. The first‐order (unifactorial) structure represents 23.14% of the common variance (Table 3).

Also, from a statistical perspective, the MHLaSS‐B can configure a multifactor structure. Double‐factor structure seems conceivable since it has theoretical justification (items that assess literacy and items that assess stigma), but a structure forced in a two‐factor model explains only 17.787% of the variance. The independence of the items in two possible MHLaSS‐B subscales was studied using inter‐item correlations. All correlations were positive and significant (significance level *α* = 0.01) and varied between lower (*r* = 0.12) and higher (*r* = 0.55) values, respectively, and the corrected item‐total correlation ranged between 0.40 and 0.56. However, considering that this is a pilot study and that the two‐factor model presents a lower value of total explained variance than the single‐factor structure, we opted for the conservative decision of maintaining the original MHLaSS‐B single‐factor structure, as recommended by some authors (Marôco et al. 2014).

### 3.5 MHLaSS‐B Reliability

Internal consistency calculated by Cronbach's *alpha* coefficient (*α* = 0.702) indicates acceptable reliability for the MHLaSS‐B and a reliable structure. All items obtained values > 0.5 except for item 27 (Table 3). Satisfactory reproducibility was indicated by the acceptable test–retest reliability (0.72), measured by the intraclass coefficient.

## 4 Discussion

This research was the first attempt to validate the instrument MHLaSS‐B to create a new bilingual version (Portuguese and Spanish) and to test the psychometric properties. The major strength of this study is that MHLaSS‐B measures simultaneously mental health literacy and stigma, which is novel and an important achievement, for clinical practice. As stated, the clinical significance of the stigma of mental illness remains an important and growing problem; therefore, there is a need for valid and reliable instruments to measure the phenomenon, both from the point of view of the patient, health professionals and students (Munawar et al. 2022).

The sample is mostly feminine, following the current gender ratio of Portuguese and Spanish students in higher education (Nogueira et al. 2022), but yet our sample is slightly older (*M* = 26.2; SD = 8.5). The majority show *High literacy* and *Low Stigma* (*M* = 23.4; SD = 3.2) revealing an important aspect of the student's sample. Study results of high knowledge and low stigma (scores between 23 and 28) reveal a good level of literacy about mental illness. Senior students show fewer stigmatisation attitudes compared with freshmen.

A significant negative correlation was found between the academic level (years course level) of study and stigma. This result is probably related to the knowledge acquired during the curricular units and due to internship and clinical teaching, where students interact with several people with mental illness in different contexts of hospitalisation. These results, in line with previous studies, show that the stigma tends to decrease along the progression of theoretical and practical training (Mackert et al. 2019; Munawar et al. 2022). Querido and colleagues, in a similar sample, also found identical results (Querido, Tomás, and Carvalho 2016). Throughout the academic path, students increase their contact with different contexts of care, which includes dealing with different patients and pathologies, including mental illnesses, contributing to diminishing the prejudice and stigma towards mental illnesses (Chang et al. 2017). However, in a recent study, authors discuss that qualifications and experience in mental health do not reduce the stigma associated with mental illness (Ocho et al. 2023) which is remarkable, once these findings input colleges' responsibilities in addressing new approaches to promote MHL among students and health professionals to achieve a major transformation.

Additionally, culture shapes many aspects of mental health. Some heritage culture labels mental illness as taboo, leading to perceiving mental illness as shameful, resulting in stigmatisation, and limiting the pathways of seeking help (Kwegyir Tsiboe, Raghuraman, and Marshall 2024). Thus, the cultural influences on mental health must be considered and addressed when dealing with perceptions and beliefs about persons with mental illness. Similarly, there is a need for the destigmatisation of attitudes and gender‐specific beliefs related to mental illness (Wills et al. 2020) by the whole society, but especially among healthcare students. Thus, the first step to avoiding negative stereotypes among doctors, social workers and nurses is to eradicate false myths about mental illness right from the academic stage.

As mentioned, training in the field of psychiatry reduces stigmatising attitudes (Mackert et al. 2019; Munawar et al. 2022). As future health professionals, all students must be aware that stigmatisation can intensify an individual's feeling of rejection and incompetence and can be adverse to treatment‐seeking and adherence behaviours. Also, students must learn how to contact and manage care with compassion and neutrality, and without prejudices or stigma for all persons (Samari et al. 2018).

Because stigma is correlated to low literacy (Simões de Almeida et al. 2023), the MHLaSS‐B may become a useful pedagogical instrument as it allows assessing the transfer of knowledge about stigma and mental health literacy among students throughout the course, and permits the identification of gaps in curricular unit contents; can be used to increase awareness about one's prejudices and to normalise destigmatise care of patients with mental illnesses.

The MHLaSS‐B's intercultural adaptation and psychometric properties are globally acceptable (Marôco et al. 2014), indicating that the scale allows, simultaneously, the assessment of mental health literacy and stigma in both languages. The construct validity shows psychometric quality, nevertheless being a pilot study, and we believe that the MHLaSS‐B robustness' can be improved in further investigations. Even though the variance could be considered low. The literature recommends not interpreting the cumulative variance explained as a single/unique indicator. Future statistical analyses, such as the Parallel Analysis, Comparative Fit Index (CFI) or RMSEA, must be done in MHLaSS‐B to support plausible representation of the underlying structure of the observed variables (Ferrando and Lorenzo‐Seva 2018; Watkins 2018). Parallel Analysis would help in deciding how many factors or components to retain in factor analysis, while RMSEA assesses the goodness‐of‐fit of a structural equation model. Both techniques contribute to the rigorous evaluation of statistical models and the identification of meaningful patterns in data (Watkins 2018).

From a statistical perspective finding, a MHLaSS‐B single‐factor structure was surprising, once a double‐factor structure seems plausible and has theoretical justification because the scale measures two concepts simultaneously (literacy and stigma). The two‐factor structure is well supported by the results that display the independence of the items in two possible MHLaSS‐B subscales, showing positive and significant correlations. Nevertheless, the two‐factor model in this study shows poorly explained variance than the one‐factor structure. Then, because this is a pilot study and no Confirmatory Factor Analysis (CFA) was performed to assess the quality of the factor structure, we made conservative decision to maintain the original structure as recommended by some authors (Marôco et al. 2014). The two‐step approach, EFA followed by CFA, is beneficial for scale development and when the factor structure is not well understood. Also, CFA is designed to confirm or test predefined hypotheses or theories where researchers specify in advance the number of latent factors and the pattern of relationships between the latent factors and the observed variables. In other words, researchers have specific expectations about the relationships or patterns in their data based on existing theories or prior research, and they use statistical methods to assess whether the data supports these expectations or not (Ferrando and Lorenzo‐Seva 2018; Watkins 2018). However, in the present study performing CFA was limited, due to the time limit and the need for a larger sample size for more reliable estimates. Thus, we propose future investigations with a larger sample and additional statistic tests (CFA) to refine and to have a robust pilot study results.

The Cronbach's *alpha* coefficient (*α* = 0.702) indicates that the MHLaSS‐B structure's internal consistency is acceptable and reliable, making it suitable for use. Because we found no studies that assess the same construct as MHLaSS‐B, comparing the alpha value between instruments was not possible (Ferrando and Lorenzo‐Seva 2018).

Considering the negative correlation between close contact of people with mental disorders and stigma in this study, the student's curriculum could focus on creating a closer contact experience between students and people with mental disorders before clinical placements. Additionally, schools could also review their preparation methods for students attending clinical placements and include managing students' expectations of the institution they would be attending for their clinical placements and the types of patients they would be interacting with as defended by several authors (Richards, O'Connell, and Dickinson 2023). This study also showed that stigma towards mental disorders varies based on their type. Possibly, schools could effectively reduce stigma towards people with mental disorders by addressing identified misconceptions. For example, addressing some specific mental health problems separately (people with depression or people with schizophrenia) rather than addressing it as a single concept, that is, people with mental disorders (Chang et al. 2017).

Finally, given the scarcity of validated instruments (Portuguese and Spanish populations) to assess literacy and stigma in mental health, we believe that MHLaSS‐B is a useful tool for health workers, higher education institutions and student education to implement widen anti‐stigma programmes (Gallego et al. 2020).

### 4.1 Limitation and Suggestions

One of the limitations of this study is related sample consisted of only nursing students. Second, future research recommendations must include confirmatory analyses to assess the quality of the factor structure by statistically testing the significance of the overall model and relationships among the items and scale (goodness‐of‐fit) with a new sample. Also, concurrent or discriminant validity with other published measures and a test–retest measure for reliability and to test stability over time must be performed. Further investigations in other populations can also improve MHLaSS‐B to enhance the generalisability of the results. Additionally, it would be valuable to consider cultural factors that may impact stigma and MHL, in future research.

## 5 Conclusions

The Mental Health Literacy and Stigma Scale‐*Bilingual* version (Spanish and Portuguese) comprises a set of items that assess Literacy and Stigma in two different languages. The MHLaSS‐B content validity and reliability tests were performed showing psychometric quality, nevertheless being a pilot study. We believe that the MHLaSS‐B robustness' can be improved in further investigations as a promising tool with several potential applications for healthcare settings. Thus, MHLaSS‐B is a reliable, adaptable instrument that is now available and it can be used in investigation, teaching and clinical practice. Having the MHLaSS‐B available allows health workers, students, nurses and researchers of Portugal and Spanish to self‐assess their stigma and to evaluate healthcare interventions established with their patients.

## 6 Implications for Practice

Considering the negative correlation between close contact of people with mental disorders and stigma in this study, the curriculum must focus on creating a closer contact experience between students and people with mental disorders before clinical placements. Additionally, schools could also review their preparation methods for students attending clinical training and include managing students' expectations of the institution they would be attending for their clinical placements and the types of patients they would be interacting with. MHLaSS‐B can be used to increase awareness about one's prejudices and to normalise and destigmatise care of patients with mental illnesses. Higher Education Institutions have the responsibility and must be prepared to effectively reduce stigma towards people with mental disorders by addressing identified misconceptions about mental disorders in their course syllabus.

## Author Contributions

All authors agree with the final version of the manuscript and the order in which the author names appear. This manuscript has not been published or submitted for publication in any other journal.

## Ethics Statement

The study protocol was registered and approved (P07/2020 ESECVP‐AT) by the Nursing School Ethics Committee Board involved and the authorisation to use and translate the scale was obtained.

## Conflicts of Interest

The authors declare no conflicts of interest.

## Data Availability

The data that support the findings of this study are available from the corresponding author upon reasonable request.
